# Prediction of Soluble-Solid Content in Citrus Fruit Using Visible–Near-Infrared Hyperspectral Imaging Based on Effective-Wavelength Selection Algorithm

**DOI:** 10.3390/s24051512

**Published:** 2024-02-26

**Authors:** Min-Jee Kim, Woo-Hyeong Yu, Doo-Jin Song, Seung-Woo Chun, Moon S. Kim, Ahyeong Lee, Giyoung Kim, Beom-Soo Shin, Changyeun Mo

**Affiliations:** 1 Agriculture and Life Sciences Research Institute, Kangwon National University, Chuncheon 24341, Republic of Korea; kim91618@kangwon.ac.kr; 2 Department of Biosystems Engineering, College of Agriculture and Life Sciences, Kangwon National University, Chuncheon 24341, Republic of Korea; hhh990604@kangwon.ac.kr; 3 Interdisciplinary Program in Smart Agriculture, Kangwon National University, Chuncheon 24341, Republic of Korea; doojin0463@kangwon.ac.kr (D.-J.S.); weez96@kangwon.ac.kr (S.-W.C.); 4 Environmental Microbial and Food Safety Laboratory, Agricultural Research Service, U.S. Department of Agriculture, Beltsville, MD 20705, USA; moon.kim@usda.gov; 5 Department of Agricultural Engineering, National Institute of Agricultural Sciences, Jeonju 54875, Republic of Korea; lay117@korea.kr; 6 Protected Horticulture Research Institute, National Institute of Horticultural and Herbal Science, Haman 52054, Republic of Korea; giyoung@korea.kr

**Keywords:** hyperspectral imaging, soluble solid content, citrus fruit, partial least-squares regression, effective-wavelength selection

## Abstract

Citrus fruits were sorted based on external qualities, such as size, weight, and color, and internal qualities, such as soluble solid content (SSC), acidity, and firmness. Visible and near-infrared (VNIR) hyperspectral imaging techniques were used as rapid and nondestructive techniques for determining the internal quality of fruits. The applicability of the VNIR hyperspectral imaging technique for predicting the SSC in citrus fruits was evaluated in this study. A VNIR hyperspectral imaging system with a wavelength range of 400–1000 nm and 100 W light source was used to acquire hyperspectral images from citrus fruits in two orientations (i.e., stem and calyx ends). The SSC prediction model was developed using partial least-squares regression (PLSR). Spectrum preprocessing, effective wavelength selection through competitive adaptive reweighted sampling (CARS), and outlier detection were used to improve the model performance. The performance of each model was evaluated using the coefficient of determination (R^2^) and root mean square error (RMSE). In the present study, the PLSR model was developed using only a citrus cultivar. The SSC prediction CARS-PLSR model with outliers removed exhibited R^2^ and RMSE values of approximatively 0.75 and 0.56 °Brix, respectively. The results of this study are expected to be useful in similar fields such as agricultural and food post-harvest management, as well as in the development of an online system for determining the SSC of citrus fruits.

## 1. Introduction

Fruit quality, including external qualities, such as color and size, and internal qualities, such as soluble solid content (SSC), acidity, and firmness, are basic factors that influence consumers when purchasing fruit [1]. Consumers prefer to purchase fruits of high internal quality rather than simply focusing on external quality. Citrus fruits are rich in nutrients and vitamin C and are among the most popular fruits with increasing consumption [1,2,3]. Citrus fruits are non-respiratory fruits; therefore, to improve industrial competitiveness and profitability, they should be harvested at an appropriate ripeness [3]. Citrus fruits have a high correlation between ripeness and SSC, and consumers prefer sweet fruits; therefore, the prediction of SSC for citrus fruits is necessary [2,4].

Traditional SSC measurement methods are unsuitable for determining the quality of fruits in high demand, such as citrus fruits, because these methods are destructive and time-consuming [1]. Therefore, visible and near-infrared (VNIR) and near-infrared (NIR) spectroscopy techniques, which can non-destructively determine fruit quality in a short time, are widely used to measure SSCs [2,5,6,7]. Spectroscopic techniques for determining fruit quality are suitable for analyzing compounds containing rich information on C-H, C-O, O-H, and N-H vibrational absorption in the VNIR- and NIR-wavelength spectra [7,8]. According to previous studies, citrus SSC predictions show that their transmittance mode is marginally higher than their reflectance mode for VNIR wavelengths [7]. The SSC prediction of citrus fruits using the transmittance mode is less sensitive to the peel thickness characteristics of the fruit and can provide high prediction performance. However, regardless of the measurement mode, the SSC prediction may be unstable because spectroscopy measures only a limited area of one point without providing information about the spatial area [2]. Since SSC is distributed differently depending on the area in a fruit, spatial information is needed to provide reliable information.

Hyperspectral imaging (HSI) is a technique that integrates the reflection spectrum of the sample and spatial information of the image, providing three-dimensional image data which can be used to estimate the internal characteristics of a fruit [2,7,9,10,11]. Owing to the more comprehensive information obtained by HSI, it is widely applied for SSC detection in several fruits such as citrus fruits, apples, grapes, and peaches [2,7,9,10,11,12]. Zhao et al. [11] predicted the SSC of grapes using hyperspectral imaging and showed excellent results with the coefficient of determination of R^2^ and root mean square error (RMSE) of 0.88 and 0.92 °Brix, respectively. Riccioli et al. [12] evaluated HSI to predict the SSC of oranges and presented a model with RMSE prediction performance of 0.86%. However, HSI produces large amounts of data, and data overlap between spectral bands may occur. These disadvantages can reduce the robustness of a model for an internal fruit-quality prediction system [10]. Therefore, to develop a robust SSC prediction model, characteristic data must be selected from hyperspectral image data. Previous studies have reported that a high-performance model can be developed by selecting characteristic wavelengths from hyperspectral image data based on the successive projection algorithm, competitive adaptive reweighted sampling (CARS), and uninformative variable elimination [1,2,9,13,14]. Moreover, effective wavelength selection using the CARS algorithm is effective for developing an SSC prediction model [2,7]. Additionally, data outliers occur during the hyperspectral image measurement, and data extraction processes and model performance can be improved by detecting and removing these outliers [2,15,16].

Therefore, this study aims to improve the prediction accuracy of citrus fruit SSC using hyperspectral image data. Specifically, the hyperspectral characteristics of citrus were investigated, the performance of a citrus SSC prediction model using an effective-wavelength selection algorithm and outlier detection was determined, and the robust models were built to determine the optimal citrus SSC prediction model.

## 2. Materials and Methods

### 2.1. Sample Preparation

A total of 324 citrus fruits *(citrus unshiu. Marcow*) were harvested from 6 October 2022 to 30 November 2022 at the Citrus Research Station in Jeju, Republic of Korea. The citrus fruits were classified according to harvest time, and the number of samples by harvest date is shown in Table 1. All the samples were stored within 8 h at 20 °C and 60% relative humidity before the acquisition of hyperspectral images to reduce the effect of temperature on the fruits [17].

### 2.2. Soluble Solid Content (SSC) Measurements

The pulp of each citrus fruit was juiced to measure the SSC, which was used as a reference for the spectrum. The SSC of each citrus sample was measured using a digital refractometer (PAL-3, ATAGO, Tokyo, Japan) immediately after HSI collection. The digital refractometer had a measuring accuracy of ±0.1 °Brix over a range of 0–93 °Brix.

### 2.3. Hyperspectral Imaging System

**Figure 1:**
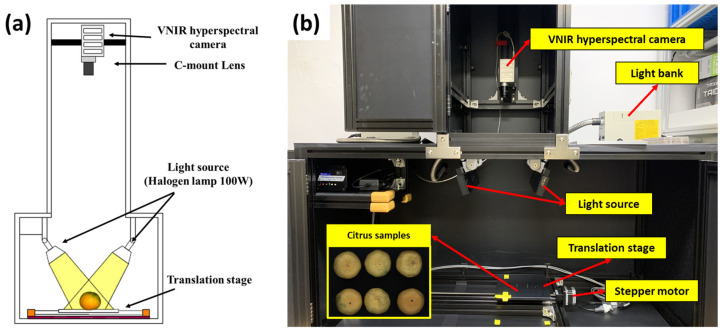
(**a**) Schematic of VNIR hyperspectral imaging system and (**b**) constructed hyperspectral imaging system.

Hyperspectral images of citrus fruits were obtained using an HSI system, which comprised an acquisition unit for HSI, two light sources, and a sample translation unit using a stepper motor (Figure 1). A 12-bit line-scan hyperspectral camera (micro HSI™ 410 Hyperspectral Sensor, Corning Inc., Corning, NY, USA) was used for the HSI of the citrus fruits at spatial and spectral resolutions of 1160 pixels and 2 nm, respectively, in the VNIR wavelength range of 400–1000 nm. Hyperspectral images of 300 bands were obtained. The horizontal and vertical axes of each line-scan image included the respective spatial and spectral information. Two 100 W quartz tungsten halogen (QTH) line lights were used as the light source and were fixed at 15° (zenith angle) to provide diffuse and well-distributed illumination. The vertical distance between the citrus sample and hyperspectral camera was set to 545 mm to avoid light saturation. The exposure time was set to 9 ms, and 400 lines were measured with the step interval of the translation stage set to 1 mm.

### 2.4. Hyperspectral Image Collection and Extraction

Two 100 W QTH line lights were turned on for 1 h prior to the measurement to stabilize the light source for uniform irradiation. The experiment was carried out in a dark room to minimize noise in the spectrum during spectral measurements. The exposure time was set to 9 ms, and 400 lines were measured with a moving step interval of 1 mm to collect the hyperspectral images of the samples. Hyperspectral images were obtained for two orientations (calyx and blossom-end sides) of each citrus sample. In total, 648 HSI data points were obtained. Dark and white reference images were obtained to correct the noise generated by the device and scattered light in the citrus sample hyperspectral images [18]. Dark reference images were obtained without light source exposure, and white reference images were obtained using diffuse reflectance standards (Labsphere, North Sutton, NH, USA). The hyperspectral images of the citrus samples were calibrated via Equation (1) using dark and white reference images [19].

(1) $$I\left(i\right)=\left(I_{s}\left(i\right)-D\left(i\right)\right)/\left(I_{w}\left(i\right)-D\left(i\right)\right)$$

where *I* is the corrected relative hyperspectral image, *I_s_* is the sample hyperspectral image, *I_w_* is the hyperspectral image of the white reference plate, and *D* is the hyperspectral image of the dark reference plate at the *i*th wavelength.

Hyperspectral data for each citrus sample were extracted from the calibrated images of the regions of interest (ROIs). The hyperspectral images of the citrus samples were separated from the background based on the reflectance value at a specific wavelength using a threshold method [20]. For the hyperspectral images of the segmented citrus samples, ROI regions were selected for each sample, and hyperspectral data were extracted. The average spectrum was calculated as one reflectance spectrum averaged over all the pixels within the ROI of each sample. Hyperspectral images were extracted using MATLAB (version R2020a, MathWorks, Natick, MA, USA).

### 2.5. Spectral Preprocessing

Spectral shape distortion, light scattering, and noise components that may arise from the external environment can affect the identification of the main spectrum [1,6,21]. In this study, the performances of the citrus fruit SSC prediction models were compared by applying various spectral preprocessing methods that can remove the noise caused by different external factors. The preprocessing of the reflectance spectra included smoothing with a moving average, first-order derivatives (Savitzky–Golay), maximum normalization, mean normalization, range normalization, the standard normal variate (SNV), and multiplicative scatter correction (MSC). Smoothing, including the moving average, can improve the signal-to-noise ratio by removing the spectral random noise that may occur from the device [6]. The moving average operates by calculating the average over a specified window of width, smoothing the trend by shifting the data window, and computing moving average values to reduce periodicity. First-order derivative preprocessing removes the baseline shift and increases spectral resolution [22,23]. Smoothing and first-order derivatives were analyzed by setting the gap size to 6 nm. Normalization minimizes the errors caused by the sample preparation step [21,22]. SNV and MSC preprocessing are commonly used when measuring solid samples and can correct the spectral errors caused by light scattering [1]. These spectral preprocessing treatments were conducted using Unscrambler X (Ver. 10.4, CAMO Software, Oslo, Norway).

### 2.6. Effective Wavelength Selection Using Competitive Adaptive Reweighted Sampling

Effective wavelength selection is the method of selecting specific wavelengths related to the target component and excluding some spectra containing wavelengths or noise that are not related to the target component, thereby reducing the amount of data that must be collected and processed and improve the model performance. In this study, a specific wavelength was selected for the algorithm proposed by Li et al. [24]. CARS is a variable selection strategy in partial least-squares (PLS) models that selects variables with large absolute values of regression coefficients and eliminates variables with small weights [9]. CARS determines a specific wavelength in four steps: Monte Carlo sampling, enforced wavelength selection through an exponentially decreasing function, competitive wavelength reduction through adaptive reweighted sampling, and utilization of the root mean square error of cross-validation (RMSECV) of each subset [9,24]. The number of Monte Carlo samplings was set to 100, and the subset with the lowest RMSECV value was determined to be the optimal variable subset. The CARS algorithm was implemented in MATLAB (version R2020a, MathWorks, Natick, MA, USA).

### 2.7. Outlier Detection

Outlier values may occur in some spectra due to input errors, sensor malfunctions, sample deterioration, and interface errors [6]. Outlier detection is an important step for identifying atypical observations in the training set. The Monte Carlo outlier detection algorithm proposed by Cao et al. is used to remove the outlier datasets [25]. Monte Carlo outlier detection uses the distribution, mean, and standard deviation of the prediction errors to detect outliers [26]. To improve the performance of the developed model, 17 hyperspectral image datasets that showed different results in the spectra were removed from a total of 648 datasets. Outliers were detected using MATLAB (version R2020a, MathWorks, Natick, MA, USA).

### 2.8. Development of Multivariate Model

The partial least-squares regression (PLSR) model is a robust algorithm for analyzing spectral data because it is insensitive to collinear variables and tolerant of numerous variables [1]. To maximize the covariance between x (spectrum) and y (SSC), the PLSR algorithm constructs an orthogonal factor set of 20 latent variables extracted from the original wavelengths. The equation of the PLSR model is shown in Equation (2) [7]. Each model is evaluated against a calibration dataset. Leave-one-out cross-validation (LOOCV) was adopted as the validation method to determine the optimal parameters of the PLSR and verify the predictive performance of the PLSR model on the calibration set. The optimal factors were obtained by minimizing the RMSE of the cross-validation. Table 2 presents the data used to develop the model. The calibration and verification datasets (predictions) were randomly classified at a ratio of 7:3. PLSR models were developed based on the full-wavelength spectra and effective wavelength bands selected by CARS. The PLSR model was generated using Unscrambler X (Ver. 10.4, CAMO Software, Oslo, Norway).

(2) $$X=TP^{T}+E,Y=UQ^{T}+F,U=TB+H$$

where *X* is an independent variable (spectral matrix); *U* is a score matrix that describes the dependent variable *Y*; *P* is an eigenvalue matrix of the independent variable; *Q* is an eigenvalue matrix of the dependent variable; *E*, *F*, and *H* are residual matrices; and *B* is a regression coefficient of PLSR.

The actual SSC in the citrus fruits was compared with those predicted from the calibration (cross-validation) or independent validation datasets using the PLSR models. The performance of each model was evaluated by estimating the coefficient of determination of the calibration set (R_c_^2^), the cross-validation set (R_v_^2^), and the prediction set (R_p_^2^), as well as root mean square error of calibration (RMSEC), cross-validation set (RMSEV), and prediction (RMSEP), in addition to the optimal factor (F) [18,27]. The model with the highest R_v_^2^ and lowest RMSEV values was selected as the optimal model.

## 3. Results and Discussion

### 3.1. Internal Quality of Citrus Fruits

A summary of the minimum, maximum, mean, and standard deviation (STD.) of the SSC of the 324 citrus fruits is shown in Table 3. The SSC of the citrus fruits ranged from 7.40 to 12.50 °Brix, with a mean value of 9.85 °Brix. The SSC of the calibration and prediction datasets ranged from 7.40 to 12.50 °Brix and from 7.50 to 12.50 °Brix, with standard deviations of 1.07 °Brix and 1.10 °Brix, respectively. Citrus fruits classified according to harvest time had an average SSC of 9.05 °Brix and 8.97 °Brix in Stage 1 and Stage 2, respectively, and no significant difference was observed in the SSC. The mean of SSC increased from Stage 2 to Stage 6. The mean SSC based on the harvest time changed from 8.97 °Brix to 11.34 °Brix, and the minimum value increased significantly.

### 3.2. Spectra Features

**Figure 2:**
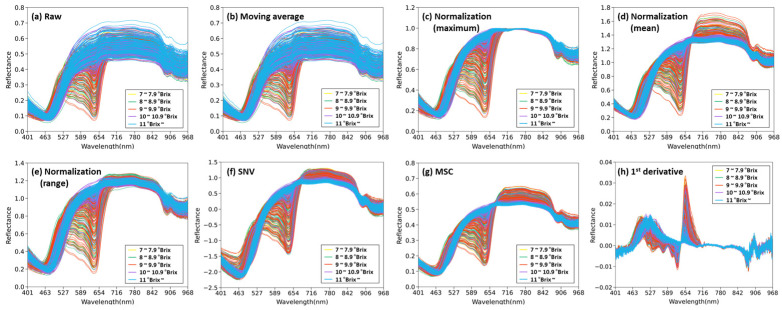
(**a**) Raw reflection spectra of citrus samples and spectra with major preprocessing: (**b**) moving average, (**c**) maximum normalization, (**d**) mean normalization, (**e**) range normalization, (**f**) SNV, (**g**) MSC, and (**h**) Savitzky–Golay first-order derivatives.

The raw reflection spectra of the citrus fruits and the reflection spectrum with the main preprocessing applied in the 400–1000 nm wavelength band are shown in Figure 2. The spectrum of the citrus fruits had negative peaks at approximately 470, 640, and 880 nm and a positive peak at 912 nm. The peak at approximately 640 nm flattened as the SSC increased. In the wavelength band below 460 nm, the negative peak appeared sharper as the SSC increased. When normalization, SNV, and MSC preprocessing (Figure 2c–g) were applied, the peak at 912 nm appeared more clearly. When mean normalization, SNV, and MSC preprocessing were applied, the lower the SSC was, the steeper the slope of the spectrum was at approximately 680–702 nm, and the wider the spectral reflectance range was at approximately 680–860 nm. When Savitzky–Golay first-order derivatives were used, strong spectral absorption peaks appeared at approximately 521, 620, 672, 792, 880, 900, and 955 nm (Figure 2h). VNIR spectra contain abundant information about the O-H, C-H, and N-H vibration absorptions [7]. The reflectance between 500 and 700 nm is caused by chlorophyll, carotenoids, and anthocyanins [28]. Fruits are composed of approximately 80% moisture, and most of the moisture absorption bands appear strongly between 960–990 nm, which are caused by O-H overtones [29]. The absorption peak at 680 nm is related to chlorophyll, and that at 960 nm is related to the second overtone of the O–H bond in the SSC [30]. In contrast, the absorption peak at approximately 948 nm is related to the decreasing sugar content [29].

### 3.3. Effective Wavelength Selection by CARS

**Figure 3:**
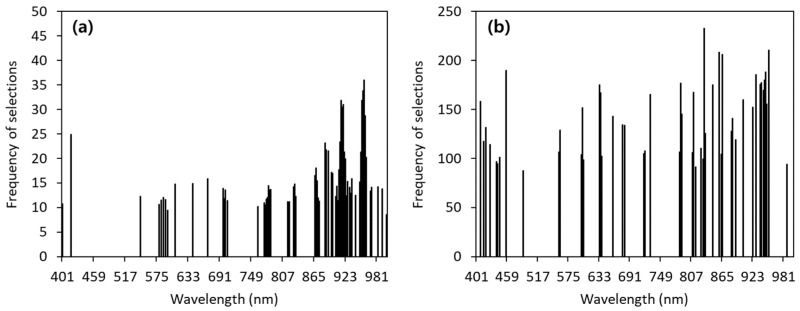
Effective wavelength selected by CARS algorithm: (**a**) when moving average preprocessing is applied to the original spectrum, (**b**) when outliers are removed and first-order differential preprocessing is applied.

A CARS algorithm was used to select the effective wavelengths based on hyperspectral data from 400 to 1000 nm. The full hyperspectral data and SSC values of the citrus samples in the calibration dataset were used as inputs, and the RMSECV was analyzed to select the effective wavelength (Table S1 in the Supplementary Materials). Effective wavelengths were extracted from 40–69 bands out of 300. Using CARS, the number of effective wavelengths was reduced to approximately 13.3%–23.1% of the 300 bands. Figure 3a,b show the wavelengths extracted from the original data and data with the outliers removed. Figure 3a is the effective wavelength of data to which moving average preprocessing was applied, and Figure 3b is the effective wavelength selected when first-order differential preprocessing was applied after removing the outliers. Effective wavelength selection can remove collinearity, redundancy, and noise in the initial spectrum, consequently enhancing performance in comparison to models based on the full spectrum [31]. The most effective wavelengths were 575–700 nm and 800–1000 nm. In addition, wavelengths between 400 and 500 nm were used. Previous studies have reported that the spectral features associated with SSC in VNIR images include the fourth overtone of CH_2_ stretching at approximately 740–780 nm, the third overtone of the C-H stretching band at approximately 900 nm, and the O-H bonding band at approximately 840 nm [2]. Tian et al. [1] reported that the effective wavelength was selected through CARS to develop a citrus SSC prediction model, and as a result, the effective wavelength was selected around 800–900 nm. The selected effective wavelengths were used to develop the citrus fruit SSC prediction model.

### 3.4. Prediction Model of Citrus Fruit SSC

A PLSR model was developed to compare the performance of the citrus fruit SSC prediction model based on effective wavelength selection through the CARS algorithm and outlier detection, and Table 4 shows the performance of each model. When spectral preprocessing was applied, the performance of the citrus fruit SSC prediction model improved. Overall, moving average preprocessing demonstrated superior performance in removing undesirable noise and reducing interference in the raw spectra. In the PLSR, CARS-PLSR, and PLSR models with outliers removed, moving average preprocessing showed the highest prediction performance when comparing the R_v_^2^ and RMSEV of the models for each preprocessing method. The R_v_^2^ values of the PLSR, CARS-PLSR, and PLSR models with outliers removed were 0.639, 0.654, and 0.707, respectively, and the RMSEV values were 0.640, 0.626, and 0.568 °Brix, respectively. The CARS-PLSR with outliers removed showed high SSC prediction performance compared to the other models. When the first-order derivative was applied, the R_v_^2^ and RMSEV were 0.716 and 0.551 °Brix, respectively, indicating that this was the optimal model for predicting the SSC of citrus fruits. The effective wavelength selection method and outlier detection improved the model performance. The optimal number of factors for CARS-PLSR with the outliers removed ranged between six and nine. The model performance was the best when first-order derivative preprocessing was applied, and the optimal number of factors was six.

### 3.5. Regression Coefficient of the PLSR Model

**Figure 4:**
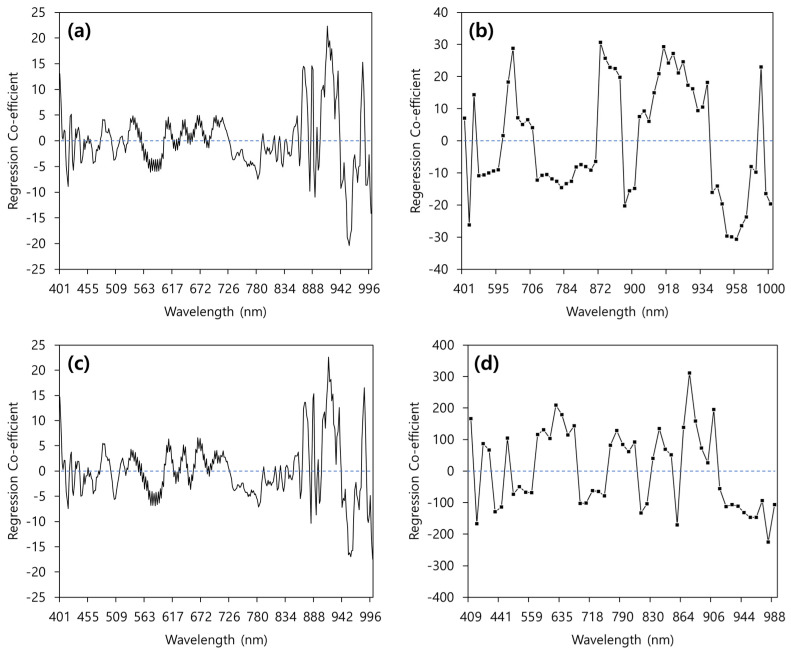
Regression coefficient for the (**a**) PLSR, (**b**) CARS-PLSR, (**c**) PLSR after removing outlier samples, and (**d**) CARS-PLSR after removing outlier samples.

Figure 4 shows the regression coefficients of the optimal model for four cases (PLSR, CARS-PLSR, PLSR with outlier samples removed, and CARS-PLSR with outlier samples removed) for predicting the SSC of citrus fruits. The regression coefficients of the PLSR model and PLSR models with outlier samples removed were similar, with only the weights differing (Figure 4a,c). The regression coefficient had large values, between 862 and 1000 nm. The regression coefficient of CARS-PLSR with moving average preprocessing showed strong peaks at 417, 670, 870, 874, 886, 892, 916–924, 954–964, 984, and 1000 nm (Figure 4b). The regression coefficient of the CARS-PLSR model, with the outlier samples removed using first-order derivative preprocessing, showed strong peaks at 409, 485, 633, 635, 860, 866, 906, 944, 946, 948, and 954 nm (Figure 4d). Liu et al. [32] developed a navel orange SSC prediction model for the development of a portable near-infrared device, and they reported that only the wavelength range of 820–950 nm was utilized, which includes the main wavelength for SSC prediction. Gomes et al. [28] reported sugar-related peaks at 740, 770, 840, 910, 960, and 984 nm. Shao et al. [33] reported that the wavelength between 850 and 950 nm can be attributed to the third overtone stretching of C-H and the second and third overtones of O-H, and that the 970–990 nm wavelength range is important for predicting the SSC of fruits. In this study, similar to previous research results, a high regression coefficient was obtained for predicting SSC content in the 850–1000 nm wavelength band.

### 3.6. Performance of the Optimal Model for Predicting SSC in Unknown Citrus Fruit Samples

**Figure 5:**
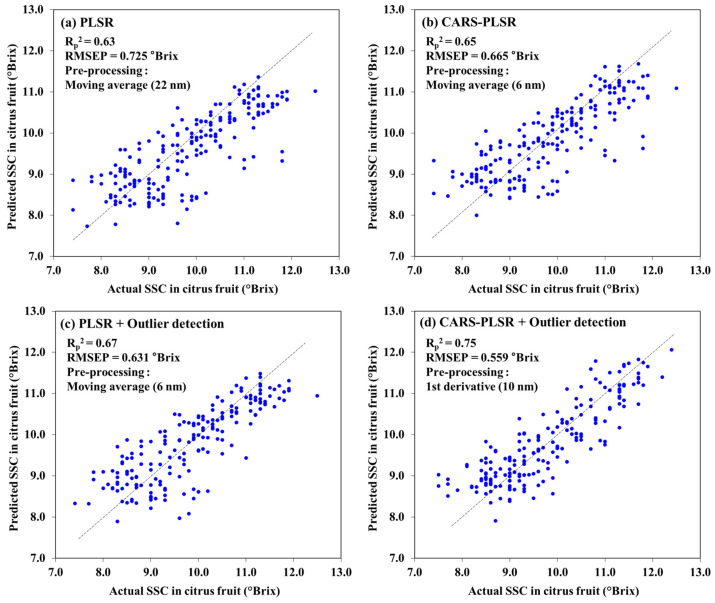
Scatter plot for citrus fruit SSC prediction results for (**a**) PLSR, (**b**) CARS-PLSR, (**c**) PLSR with outlier detection, and (**d**) CARS-PLSR with outlier detection (the dashed line is the 1:1 line).

The performance of the developed model was evaluated using a prediction dataset that was not used for the model development. Figure 5 shows the scatter plots of the predicted SSC of the citrus fruit samples based on the optimal PLSR, CARS-PLSR, PLSR with outliers removed, and CARS-PLSR with outliers removed. For the model with outlier samples removed, the R_p_^2^ and RMSEP were 0.67 and 0.631 °Brix, respectively, and this model exhibited a higher performance than the CARS-PLSR model using the effective wavelength selection algorithm. The R_p_^2^ and RMSEP values of the CARS-PLSR model were 0.65 and 0.665 °Brix, respectively. In the case of the PLSR model with effective wavelength selection and outliers removed, the R_p_^2^ and RMSEP were 0.75 and 0.559 °Brix, respectively, indicating a higher performance than other models. In previous studies, a deep learning-based the principal component analysis-back propagation (PCA-PPNN) was developed for predicting the SSC of navel oranges in the 350–1800 nm range, showing an RMSEP of 0.68 °Brix [30]. Kim et al. [34] developed a PLSR model for predicting the sugar content of *citrus unshiu*; the model was verified through a test dataset, and the performance R^2^ and RMSE of the model were 0.652 and 0.512 °Brix, respectively. Torres et al. [3] reported that the SEP and R_p_^2^ were 0.71 and 0.57, respectively, based on a model developed to predict the SSC of mandarins using a portable spectrometer based on reflection spectrum measurement. Pires et al. [4] used the SSC prediction PLS model of ‘Ortanique’ to obtain R^2^ and RMSEP values of 0.79 and 0.75%, respectively. As shown previously, citrus species result in a wide range of SSC prediction performances, depending on peel thickness. However, citrus fruit SSC prediction results for similar thicknesses showed RMSE levels ranging from 0.51 to 0.93 °Brix [1,3,4,34]. Riccioli et al. [12] developed an artificial neural network model to predict SSC of intact orange using hyperspectral images, and the R^2^ and RMSE were 0.51 and 0.86%, respectively. CARS-PLSR with outlier detection, the optimal model in this study, showed similar or a better performance than the previous results. In comparison to the findings of Riccioli et al. [12], which predicted the SSC of citrus fruits using 180 bands of hyperspectral data, this study demonstrated high accuracy with a limited number of bands.

## 4. Conclusions

This study evaluated the feasibility of using hyperspectral images as data for citrus fruit SSC prediction and developed a citrus fruit SSC prediction model based on a machine learning model. To improve the citrus fruit SSC prediction performance, an optimal spectrum preprocessing method was identified, and the performances of the prediction models through effective wavelength selection and outlier detection were compared. Effective wavelength selection and outlier detection methods were shown to be effective in improving the performance of the PLSR model for predicting citrus fruit SSC. Regarding spectral preprocessing, the moving average and first-order derivative were observed to be more powerful in predicting the citrus fruit SSC than other preprocessing methods.

In the current state of the Republic of Korea’s citrus sorting lines, the permissible range for predicted SSC errors is set at 0.5 °Brix. In the case of the CARS-PLSR model with the outlier sample removed, the R_p_^2^ value was 0.75 and the RMSEP was 0.559 °Brix, which exhibited an improvement over the original PLSR model, allowing us to build a robust model. Upon reviewing the latest technological trends (state-of-the-art), previous studies have shown that similar prediction models had RMSEP ranges from 0.51 to 0.93 °Brix. Therefore, considering the current research trends, our study’s CARS-PLSR with outlier detection model demonstrates similar accuracy in comparison to the existing technological standards for sorting machines within the Republic of Korea [35].

In addition, 51 spectra were selected as effective wavelengths using the CARS algorithm in CARS-PLSP with outlier samples removed, which was the optimal model for predicting citrus fruit SSC. The results indicated that employing CARS algorithm significantly enhanced the prediction accuracy while reducing the dimensionality.

This study confirmed that the SSC of citrus fruits can be rapidly predicted using HSI. HSI is valuable for enhancing the quality assessment of fruits as it offers comprehensive data, including the SSC of each fruit part, presented through images and spectrum data. In contrast, conventional optical sorting systems measure only a single representative value, yielding relatively limited information. The sorting systems based on hyperspectral sensors are poised to provide a robust foundation for determining the pricing of citrus based on quality evaluation. Moreover, the results of this study showed performance similar to or higher than the SSC prediction performance when using the full spectrum in previous studies through the selected effective wavelength. These findings suggest that the improved performance in predicting citrus fruit SSC through effective wavelength selection could be used as fundamental data for designing a cost-effective multispectral sensor that utilizes only the relevant wavelength band, which is more economical than a hyperspectral sensor, to accurately and rapidly predict the SSC of citrus fruits. In addition, the results of this study are expected to be useful in similar fields, such as agricultural and food post-harvest management, as well as in the development of an online system for determining the SSC of citrus fruits.

However, in the present study, the PLSR model was developed using only a citrus cultivar. Although the predicted SSC errors are within the acceptable domestic range, there is still room for improvement in refining and enhancing the model’s predictive accuracy. In future research, the utilization of various cultivars of citrus samples for the development of a more powerful prediction model and the further optimization of the proposed model through a deep learning-based model, such as an artificial neural network, backpropagation neural network, and convolutional neural network development, is intended. Additionally, the development of prediction models for various internal and external quality parameters, such as acidity, firmness, and the maturity of citrus fruits, will be further investigated in the future.

## Figures and Tables

**Table 1:** Number of samples by harvest date.

Harvest Date	6 October 2022	15 October 2022	29 October 2022	10 November 2022	19 November 2022	30 November 2022
Number of samples	74	50	50	50	50	50

**Table 2:** Datasets used to develop and validate PLSR models for predicting SSC in citrus fruits.

Index	PLSR	CARS-PLSR	PLSR + Outlier Detection	CARS-PLSR + Outlier Detection
Calibration dataset	454	454	442	442
Prediction dataset	194	194	189	189

**Table 3:** Statistical results of the SSC (°Brix) of citrus samples in the calibration and prediction sets.

Sample Set	Min. (°Brix)	Max. (°Brix)	Mean (°Brix)	STD. ^(a)^ (°Brix)
Stage 1	7.40	11.50	9.05	0.81
Stage 2	7.50	11.20	8.97	0.75
Stage 3	8.40	11.80	9.59	0.73
Stage 4	8.80	11.80	9.96	0.61
Stage 5	9.20	12.20	10.56	0.65
Stage 6	10.20	12.50	11.34	0.43
Calibration dataset	7.40	12.50	9.89	1.07
Prediction dataset	7.50	12.50	9.76	1.10
Total	7.40	12.50	9.85	1.08

^(a)^ STD: standard deviation.

**Table 4:** Results of citrus fruit SSC prediction models based on effective wavelength selection and outlier detection.

Model	Preprocessing	R_c_^2^	RMSEC (°Brix)	R_v_^2^	RMSEV (°Brix)	Optimal Factors
PLSR	Raw	0.667	0.613	0.626	0.651	11
	Moving average	0.669	0.611	0.639	0.640	13
	NOR ^(a)^ (maximum)	0.650	0.628	0.610	0.665	10
	NOR (mean)	0.644	0.634	0.605	0.669	10
	NOR (range)	0.645	0.633	0.608	0.666	10
	SNV	0.643	0.635	0.610	0.665	9
	MSC	0.614	0.660	0.581	0.689	7
	1st order derivative	0.647	0.631	0.603	0.670	5
CARS-PLSR	Raw	0.671	0.609	0.646	0.633	9
	Moving average	0.677	0.604	0.654	0.626	9
	NOR (maximum)	0.662	0.617	0.640	0.638	8
	NOR (mean)	0.668	0.612	0.646	0.634	9
	NOR (range)	0.670	0.610	0.648	0.632	9
	SNV	0.665	0.614	0.648	0.631	9
	MSC	0.641	0.637	0.619	0.657	8
	1st order derivative	0.565	0.700	0.530	0.731	10
PLSR + Outlier detection	Raw	0.736	0.536	0.704	0.569	11
	Moving average	0.738	0.534	0.707	0.568	12
	NOR (maximum)	0.720	0.552	0.682	0.589	10
	NOR (mean)	0.715	0.558	0.685	0.586	10
	NOR (range)	0.715	0.557	0.683	0.589	10
	SNV	0.710	0.562	0.681	0.591	9
	MSC	0.709	0.563	0.679	0.593	9
	1st order derivative	0.710	0.562	0.676	0.596	5
CARS-PLSR + Outlier detection	Raw	0.722	0.545	0.700	0.575	8
	Moving average	0.733	0.533	0.715	0.553	9
	NOR (maximum)	0.723	0.543	0.702	0.565	8
	NOR (mean)	0.730	0.537	0.711	0.556	7
	NOR (range)	0.707	0.559	0.688	0.578	7
	SNV	0.728	0.538	0.711	0.558	7
	MSC	0.715	0.551	0.695	0.573	7
	1st order derivative	0.745	0.522	0.716	0.551	6

^(a)^ NOR: normalization.

## Data Availability

Data are contained within the article.

## Supplementary Materials

The following supporting information can be downloaded at: https://www.mdpi.com/article/10.3390/s24051512/s1, Table S1: Effective wavelengths for each model selected with the CARS algorithm.
