# Supplementary material for: Prediction of Soluble-Solid Content in Citrus Fruit Using Visible–Near-Infrared Hyperspectral Imaging Based on Effective-Wavelength Selection Algorithm
Source: Sensors (Basel). 2024 Feb 26;24(5):1512. doi: 10.3390/s24051512 (PMC10935418; doi:10.3390/s24051512)
Supplement: Supplementary file 1 [file sensors-24-01512-s001.zip › sensors-2701210-supplementary.pdf]

Supplementary materials

Table S1. Effective wavelengths for each model selected with the CARS algorithm

Algorithms	Preprocessing	No. of selected bands	Selected bands (nm)
CARS-PLSR	Raw	69	417-427, 437, 443, 547, 581, 585, 589, 593, 597, 607, 611, 639, 668, 696, 700, 704, 764, 772, 780-784, 788, 818-820, 828-832, 862-864, 868- 876, 884-888, 892, 898-900, 906-922, 926, 930, 934, 952- 962, 970-972, 984, 990-992, 1000
	Moving average	64	401, 417, 545, 579, 583, 587, 591, 595, 609, 642, 670, 698-702, 706, 762, 774-786, 818-820, 828-832, 868-876, 886-888, 892, 898-900, 906-928, 932-936, 942, 950-962, 970-972, 984, 992, 1000
	Normalization (maximum)	51	401, 417-421, 425-427, 437, 589, 597, 639, 700, 734, 738, 782-784, 800, 808, 818-820, 826-832, 862-870, 874-876, 884-886, 900, 906-908, 912, 916-922, 926, 930, 946, 952-960, 970-972, 992
	Normalization (mean)	56	401, 415-421, 425-429, 437, 453, 543, 547, 589, 593, 597, 639, 644, 668, 672, 700, 818-820, 828-832, 862-870, 876-878, 884-888, 892, 900, 906-908, 912-922, 926, 930, 934, 952-960, 970-972, 992
	Normalization (range)	40	419, 425-427, 437, 445, 453, 461, 525, 543, 547, 593, 597, 644, 668, 734, 818-820, 830, 862-864, 868, 876, 886, 892, 900, 906, 914-922, 926, 930, 952-960, 970, 992
	SNV	36	419-421, 427, 501, 589, 593, 597, 639, 696, 700, 784, 818, 826-832, 864, 868-870, 876, 886, 900, 906-908, 918-922, 930, 938, 954-956, 960-962, 970, 980, 992
	MSC	36	419, 427, 437, 443-445, 453, 485, 491, 589, 593, 734, 808, 818, 826-832, 864-868, 876, 884, 892, 906, 912, 918-922, 930, 946, 952-956, 960-962, 970
	1st derivative	34	421, 431, 435, 455, 489, 497, 635, 716, 732, 770, 786-790, 810, 818, 832-834, 848, 856, 860, 866, 878, 890, 894, 904, 940, 946-948, 952, 960-962, 972-974
CARS-PLSR + Outlier detection	Raw	62	401, 419-421, 425-427, 437, 443, 543, 581, 585, 589, 593, 597, 607, 611, 639, 668, 672, 700, 704, 764, 772, 780-784, 818-820, 828-830, 862-864, 868-872, 876, 884-888, 898-900, 906-922, 926, 934, 952-960, 970-972, 984, 990-992, 1000
	Moving average	56	401, 417, 545, 583,587, 591, 609, 642, 670, 698, 702, 706, 774, 778, 780-786, 818-820, 828-830, 868-876, 886-888, 892, 898-900, 906-924, 928, 936, 950-962, 970-972, 984, 990-992
	Normalization (maximum)	44	401, 419, 427, 539, 589, 593, 639, 668, 672, 784, 808, 818-820, 828-830, 862-872, 876-878, 884-888, 898-900, 906, 910, 914-922, 930, 952-960, 966, 970-972, 992
	Normalization (mean)	42	401, 419, 425-427, 437, 443, 505, 585, 589, 593, 734, 804, 808, 818-820, 826-830, 862-864, 868-870, 876, 884-

			886, 900, 906, 912, 918-922, 930, 946, 952- 962, 966, 970, 992
	Normalization (range)	51	401, 419-421, 425-427, 437, 543, 585, 589, 593, 639, 664, 668, 784, 808-810, 818-820, 862-864, 868-872, 876-878, 884-888, 898-900, 906, 910-922, 930, 934, 946, 952-960, 966, 970-972, 992, 1000
	SNV	42	401, 415, 419, 425-427, 437, 503, 585, 589, 784, 804, 808-810, 818-820, 830, 862-864, 868-870, 876, 884-886, 900, 906-908, 912, 918, 920-922, 930, 946, 952-962, 966, 970, 990-992
	MSC	40	401,407, 419, 425-427, 437, 445, 453, 543, 589, 593, 597, 607, 611, 639, 668, 672, 700, 818-820, 862, 868-870, 876, 886, 900, 906, 910, 914, 918-922, 930, 946, 952-956, 960, 972, 992
	1st derivative	51	409, 415, 419, 427, 439-441, 445, 457, 489, 557-559, 599-603, 633-637, 660, 678, 682, 718-720, 730, 786-790, 810-812, 816, 826, 830-834, 848, 860, 864-866, 884-886, 892, 906, 924, 930, 938-940, 944-950, 954, 988
